# Robust Cell Detection for Large-Scale 3D Microscopy Using GPU-Accelerated Iterative Voting

**DOI:** 10.3389/fnana.2018.00028

**Published:** 2018-04-26

**Authors:** Leila Saadatifard, Louise C. Abbott, Laura Montier, Jokubas Ziburkus, David Mayerich

**Affiliations:** ^1^Department of Electrical and Computer Engineering, University of Houston, Houston, TX, United States; ^2^College of Veterinary Medicine and Biomedical Sciences, Texas A & M University, College Station, TX, United States; ^3^Department of Biology and Biochemistry, University of Houston, Houston, TX, United States

**Keywords:** cell detection, image processing, GPU, big data, microscopy, KESM

## Abstract

High-throughput imaging techniques, such as Knife-Edge Scanning Microscopy (KESM),are capable of acquiring three-dimensional whole-organ images at sub-micrometer resolution. These images are challenging to segment since they can exceed several terabytes (TB) in size, requiring extremely fast and fully automated algorithms. Staining techniques are limited to contrast agents that can be applied to large samples and imaged in a single pass. This requires maximizing the number of structures labeled in a single channel, resulting in images that are densely packed with spatial features. In this paper, we propose a three-dimensional approach for locating cells based on iterative voting. Due to the computational complexity of this algorithm, a highly efficient GPU implementation is required to make it practical on large data sets. The proposed algorithm has a limited number of input parameters and is highly parallel.

## 1. Introduction

Finding positions of cell nuclei is important for several biomedical applications, including cancer research (Dow et al., 1996), disease diagnosis (Zink et al., 2004), neurodegenerative disease research (Li et al., 2007a), and *in vitro* tracking (Merouane et al., 2015). Several cell localization methods have been explored in the past few decades. However, they are mostly limited to two dimensional datasets, and the available three-dimensional (3D) algorithms are inaccurate, slow, or difficult to automate due to common variations in cell size, shape, and proximity. Perhaps the most challenging problem to address is computation speed, which significantly impacts processing large images. Recent advances in high-throughput imaging allow researchers to acquire images of whole brains (Yuan et al., 2015; Xiong et al., 2017) containing cellular data that is difficult to segment. Processing these data sets using traditional methods is time consuming and impractical.

### 1.1. Knife edge scanning microscopy (KESM)

**Figure 1:**
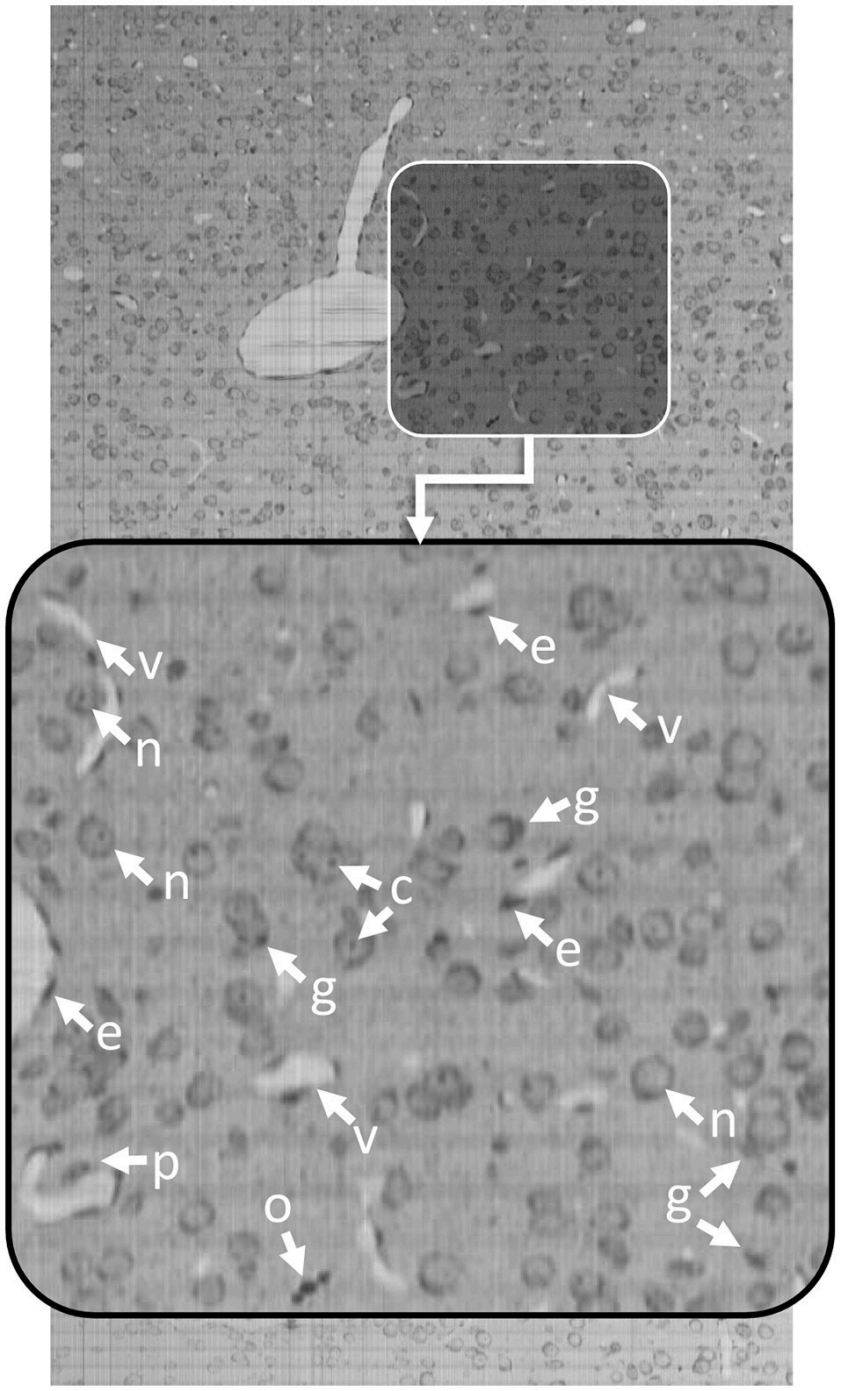
Thionine-stained mouse cortex imaged using KESM with a 1 μm section thickness. Thionine is a nucleic acid stain, labeling DNA (cell nuclei) and ribosomal RNA, which is dense within the neuron cytoplasm. The arrows indicate tissue features: endothelial cell nuclei (e), neuron nucleoli (c), glial cells (g), neuron nuclei (n), pericytes (p), and oligodendrocyte nuclei (o). The surrounding neuropil is stained a light gray, making the unstained microvessels (v) visible in 3D.

Knife-Edge Scanning Microscopy (KESM) is an optical imaging technique that allows researchers to quickly collect terabyte-scale 3D images by serially sectioning a sample (Mayerich et al., 2008) labeled using either traditional brightfield stains, such as thionine, Golgi-Cox, or hematoxylin and eosin (H&E), as well as transgenic fluorescent labels (Qi et al., 2015). KESM allows researchers to collect detailed images describing cell structure and vascular/neuronal connectivity across large (cm^3^) volumes. Since the imaging is destructive, most labeling techniques attempt to maximize the amount of information collected in a single imaging pass. Optimal stains, such as thionine, provide multiple structural features in a single channel. Thionine staining is common in neuroscience for labeling DNA and ribosomal RNA by binding to acidic proteins and nucleic acids. This label provides contrast for neurons, endothelial cells, and various glial cells (Figure 1). While thionine is not generally considered to provide three-dimensional structure, the use of KESM also provides microvascular images, which are unstained and surrounded by labeled endothelial cell nuclei (Xiong et al., 2017).

### 1.2. Previous work

Our three primary goals for cell localization in KESM images are (a) automation, (b) speed, and (c) accuracy. While accuracy is generally a priority in most algorithms, the bottleneck for large analysis is processing speed. Maximizing data throughput is critical for applying any practical segmentation algorithm to big data. Consequently, cellular detection for large data sets requires fully automated algorithms, since user interaction is impractical for data sets containing more than a few thousand cells embedded in several terabytes of raw image data. In general, our priority is to achieve segmentation throughput—including user interaction—that is comparable to the acquisition time of the original raw image. At that point, we elect to optimize accuracy.

There are several automated techniques for segmenting cell nuclei in two-dimensional images. Template matching algorithms (Chen et al., 2013; Liu et al., 2016; Zarella et al., 2017) can be reliable when the cell structure is known. These algorithms are practical in 2D since the number of orientations for non-symmetric cells is limited. The number of required template tests also increases with cell variety and size. A traditional Laplacian of Gaussian (LOG) blob detector (Marr and Hildreth, 1980) is fast but provides low accuracy in practice. However, over-segmentation using LoG filters is often a starting point for multi-step algorithms (Bjornsson et al., 2008). Contour detection (Wienert et al., 2012; Lotfollahi et al., 2017) and level set methods (Cremers et al., 2007; Dzyubachyk et al., 2010; Chinta and Wasser, 2012) require some starting point and rely on a time-consuming physical evolution algorithm. Some active contours, such as snakuscules (Thevenaz and Unser, 2008), rely on very simple optimization and mitigate the need for a seed point by relying on speed and initializing the contours in a dense grid. However, these methods require excessive data fetches and current theory doesn't extend to higher-dimensional images.

Three-dimensional techniques are available in the FARSIGHT Toolkit (Bjornsson et al., 2008), and rely on a multi-step process that binarizes the input based on graph cuts and then detects the seed points using a scale-space LoG filter. Various versions of nuclei detection rely on gradient flow tracking (Li et al., 2007b) or spectral clustering (Lou et al., 2012, 2014). Others combine local adaptive pre-processing with decomposition based on line of sight to separate apparently touching cell nuclei (Mathew et al., 2015). A graph based segmentation technique has been developed (Arz et al., 2017) for a fast and efficient binarization, and a nucleus model constructed to partition the foreground. Finally, machine learning approaches (Sommer et al., 2011) are also available and we have previously reported the use of a multi-layer perceptron for cell detection on similar data sets (Mayerich et al., 2011).

Iterative voting (Parvin et al., 2007; Han et al., 2011) is a highly robust algorithm that relies on radial symmetry. It requires minimal initial information and can be applied to cells of various sizes. However, this algorithm requires a significant amount of computation, making it generally impractical for large data sets and video. However, iterative voting can be highly parallelized for use on large data sets. GPUs are widely used to accelerate computationally expensive algorithms for volumetric datasets (Pan et al., 2008; Shi et al., 2012; Eklund et al., 2013).

In this paper, the iterative voting algorithm is used to localize cell nuclei. We describe a highly parallel 3D algorithm that can be readily extended to whole brain data sets.

## 2. Methods

Iterative voting (Parvin et al., 2007) is an automated technique based on radial symmetry that requires very little user input. We first provide an overview of the iterative voting method and propose a novel 2D GPU implementation. Using these principals, we then develop a novel 3D voting technique that takes advantage of additional optimizations that are possible in higher dimensions. Finally, we validate these methods, provide profiling results, and discuss avenues for future work.

### 2.1. Two-dimensional iterative voting

Raw images are first optionally blurred by a Gaussian filter (σ_*G*_ ≈ 2 pixels) to remove noise, creating an input image *I*. The gradient is computed to initialize three fields:

(1) $$\begin{array}{l} M=|\nabla I| \end{array}$$

(2) $$\begin{array}{l} \Theta_{0}=\tan^{-1}\left(\frac{I_{y}}{I_{x}}\right) \end{array}$$

(3) $$\begin{array}{l} V_{0}=0 \end{array}$$

where *M*(*x, y*) ∈ ℝ is the gradient magnitude, Θ_0_(*x, y*) ∈ [0, 2π) is the initial direction field specified in angular coordinates, and *V*_0_(*x, y*) ∈ ℝ is the initial vote field.

**Figure 2:**
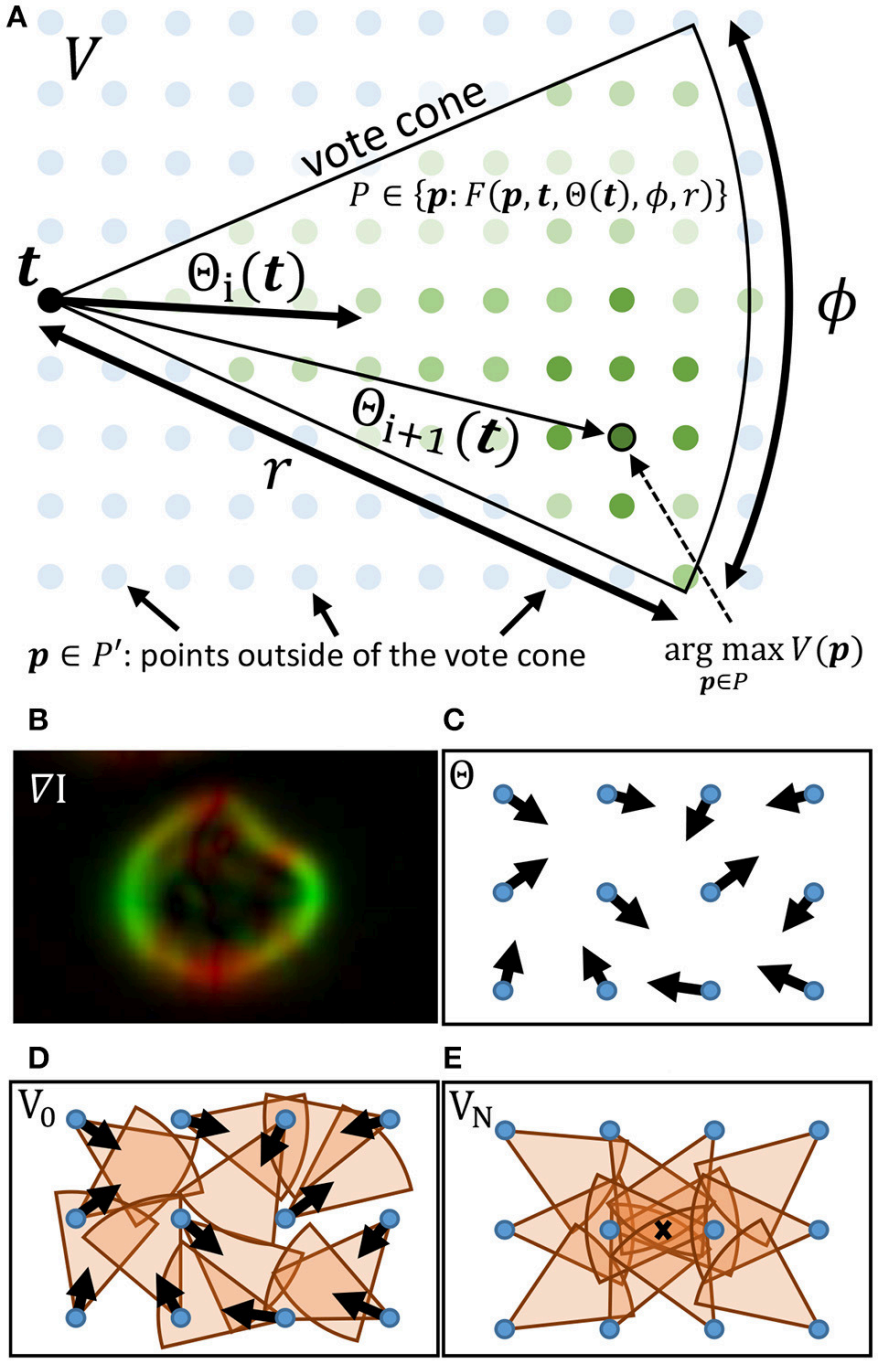
**(A)** The cone for a voter at **t** is overlayed on the vote image *V*, where green dots represent points within the vote cone that will receive votes. The orientation of the cone is given by Θ(**t**). When updating the voter direction, the maximum value of *V* within the vote cone determines the new value for Θ. **(B–E)** Important steps in the iterative voting process are also shown. **(B)** The gradient of the input image is calculated and used to initialize Θ. Cones are shown for voters in their initial state **(D)** as well as after several iterations **(E)**. The local maxima (X) indicates the cell location.

The gradient direction Θ and magnitude *M* are used to initialize a set of *T* voters consisting of a position **t** ∈ ℝ^2^, a magnitude *m*_*t*_ ∈ ℝ [where *m*_*t*_ = *M*(**t**)], and direction θ_*t, i*_ ∈ [0, 2π) which is iteratively updated such that *i* ∈ [1, *N*]. During each iteration *i*, a vote image *V*_*i*_ is initialized to zero. Each voter *t* = [1, *T*] applies a vote with weight *m*_*t*_ to all pixels within a *voting cone* oriented along θ_*t*_ with length *r* (Figure 2). The voting cone will be narrowed after each iteration by reducing ϕ. A new vote direction is determined for each voter by orienting θ_*t, i*+1_ toward the maximum value *V*_*i*_ within the voting cone. This results in a series of iteratively refined vote images *V*_*i*_ (Figure 2).

After a specified number of iterations, local maxima within the final vote image *V*_*N*_ are calculated to determine the set of *K* cell locations *C* = [**c**_1_, **c**_2_, …, **c**_*K*_]. The final vote image provides a score *s*_*k*_ = *V*_*N*_[**c**_*k*_] that can be used to infer the likelihood that **c**_*k*_ corresponds to a cell position.

**Figure 3:**
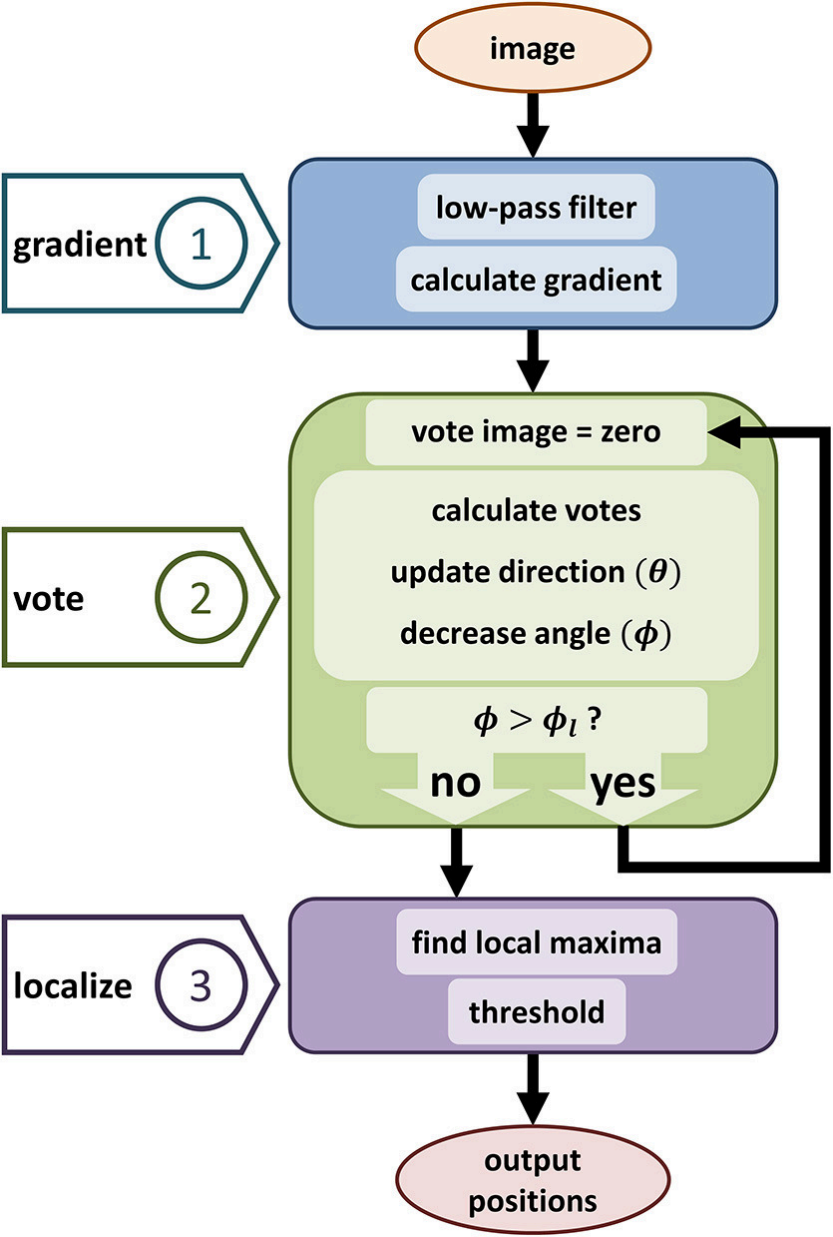
Diagram showing the proposed iterative voting algorithm steps. The first step computes the input gradient. The main step is generating vote image, the same size image as the input. This algorithm iterates through voting steps, to update voting fields and generate a converged vote image. Third step localizes cell positions by using the last vote image.

We first reformulate the iterative voting algorithm to minimize input parameters. Our proposed algorithm is outlined in Algorithm 1 and shown in Figure 3. We propose the following modifications:

- **Uniform voting grid**. Previous implementations reduce evaluation time by limiting voters to locations where *M*[*x, y*] > *m*. Our implementation assumes that all pixels as voters, which are represented using the uniform grids *M* and Θ. While this significantly increases the voter count, it removes the need for a threshold *m* and provides several advantages for parallelization (section 2.2).
- **Vote field weighting**. Previous algorithms (Parvin et al., 2007) apply a user-defined weight to each vote. We found no significant reduction in performance by eliminating this weighting.
- **Iteration count**. Previous algorithms require an initial voting angle ϕ_0_ and number of iterations *n*. We found that iterative voting is insensitive to values of $\phi_{0}>\frac{\pi }{4}$, so we use $\phi_{0}=\frac{\pi }{2}$ as a conservative starting point. The number of iterations is determined by performing a binary search until ϕ < ϕ_*t*_. The terminating condition ϕ_*t*_ is selected such that the voting angle is less than one pixel wide, at which point no new information can be extracted from the image.
- **Vote cone bounding volume**. We implement a bounding volume to limit the number of points tested for vote cone membership.

These modifications remove all input values except a cell radius estimate *r*, which is based on the magnification and resolution of the input image *I*. In the following sections, we will detail how our modified algorithm is implemented in two dimensions (Algorithm 1).

**Algorithm 1:**
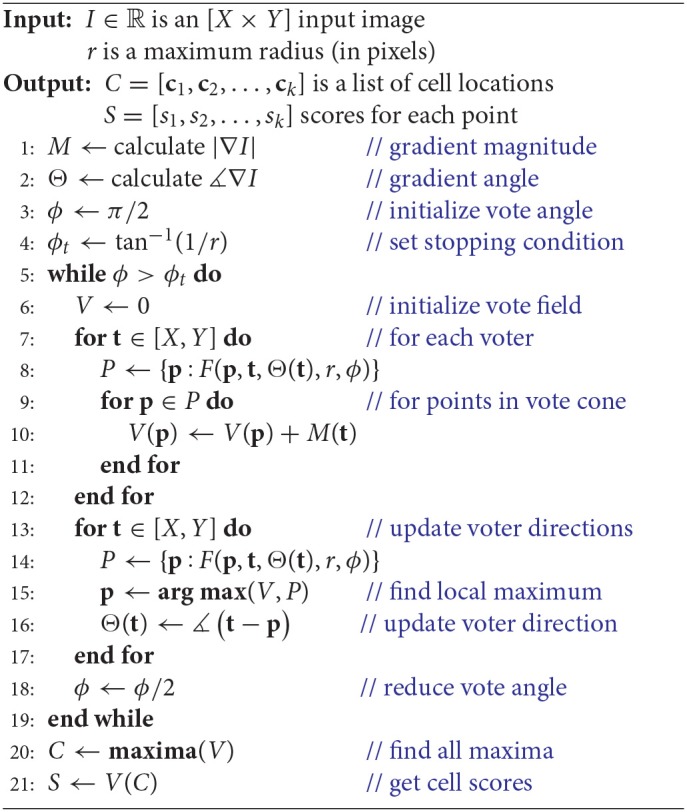
Iterative voting in two dimensions.

#### 2.1.1. Calculating vote cones

All pixels (*x, y*) ∈ [*X* × *Y*] in the input image act as voters. Since a high gradient magnitude correlates with the presence of cell boundaries, votes are weighted by *M* (Equation 1) at the voter location. Votes are applied to pixels in the vote field *V* based on their position relative to voters. A pixel at **p** in *V* will receive a vote from **t** if it lies within the *vote cone* of **t** defined by:

(4) $$\begin{array}{l} -\frac{\phi }{2}<\tan^{-1}\left(\frac{t_{y}-p_{y}}{t_{x}-p_{x}}\right)-\Theta \left(\text{t}\right)<\frac{\phi }{2} \end{array}$$

and

(5) $$\begin{array}{l} |\text{t}-\text{p}|<r \end{array}$$

where **t** is a voter position and **p** is a position in *V* that receives the vote (Figure 2). This allows us to define a membership function *F*(**p**, **t**, Θ(**t**), *r*, ϕ) ∈ 𝔹 that is true if **p** is within the voting cone of **t** (Algorithm 1, line 8).

We limit the number of pixels tested by generating a bounding volume around the vote cone. We describe this method in detail in our 3D algorithm, since the 2D case is a trivial modification of the 3D method (section 2.3).

#### 2.1.2. Calculating vote images

The vote image *V* is re-calculated every iteration (Algorithm 1, line 10), providing progressively more refined estimates of cell locations. This field can be calculated in two ways:

- **Collect Votes** - For each point **p** in the vote image *V*, find the set of voters *T* with vote cones containing **p**: *T* ∈ {**t**:*F*(**p**, **t**, Θ(**t**), *r*, ϕ)}. Finally, sum the contributions of all voters:
  (6) $$\begin{array}{l} V\left(\text{p}\right)=\underset{\text{t}\in T}{\sum }M\left(\text{t}\right) \end{array}$$
- **Project Votes** - For each voter **t**, find the set of points *P* in its vote cone: *P* ∈ {*p*:*F*(**p**, **t**, Θ(**t**), *r*)}. Finally, add the value of *M*(**t**) to *V* at each point in *P*.

By optimizing the calculation of *P* ∈ {*p*:*F*(**p**, **t**, Θ(**t**), *r*, ϕ)} and parallelizing, we demonstrate that the second case can be implemented efficiently on a GPU and is therefore expressed in Algorithm 1 (line 8). This technique saves a significant number of data fetches, providing tremendous efficiency gains when moving into higher dimensions.

#### 2.1.3. Update voter directions

The voter direction (Θ), vote angle ϕ, and *r* determine the orientation and size of the vote cone at each iteration. While the update function for ϕ is simple (Algorithm 1, line 18), the update function for Θ is based on the vote image *V* (line 16). For each voter **t**, we first find the position **p** corresponding to the maximum value of *V* within the vote cone. The new vote direction is oriented toward the location of this local maximum:

(7) $$\begin{array}{l} \Theta \left(\text{t}\right)=\tan^{-1}\frac{d_{y}}{d_{x}} \end{array}$$

where

(8) $$\begin{array}{l} \text{d}=\left(\underset{\text{p}\in P}{\text{arg max }}V\left(\text{p}\right)\right)-\text{t} \end{array}$$

#### 2.1.4. Cell localization

The vote image *V* is iteratively refined until the stopping condition ϕ < ϕ_*t*_ is reached (Algorithm 1, line 18). The final vote image is then processed to find local maxima and corresponding score values. Local maxima are selected subject to the constraint that peaks are separated by at least a distance *r*. If multiple local maxima are clustered within a distance *r*, the maximum with the highest score is maintained. A demonstration of the results of this algorithm on 2D images is provided in the Supplementary Material.

### 2.2. Data parallel implementation

**Figure 4:**
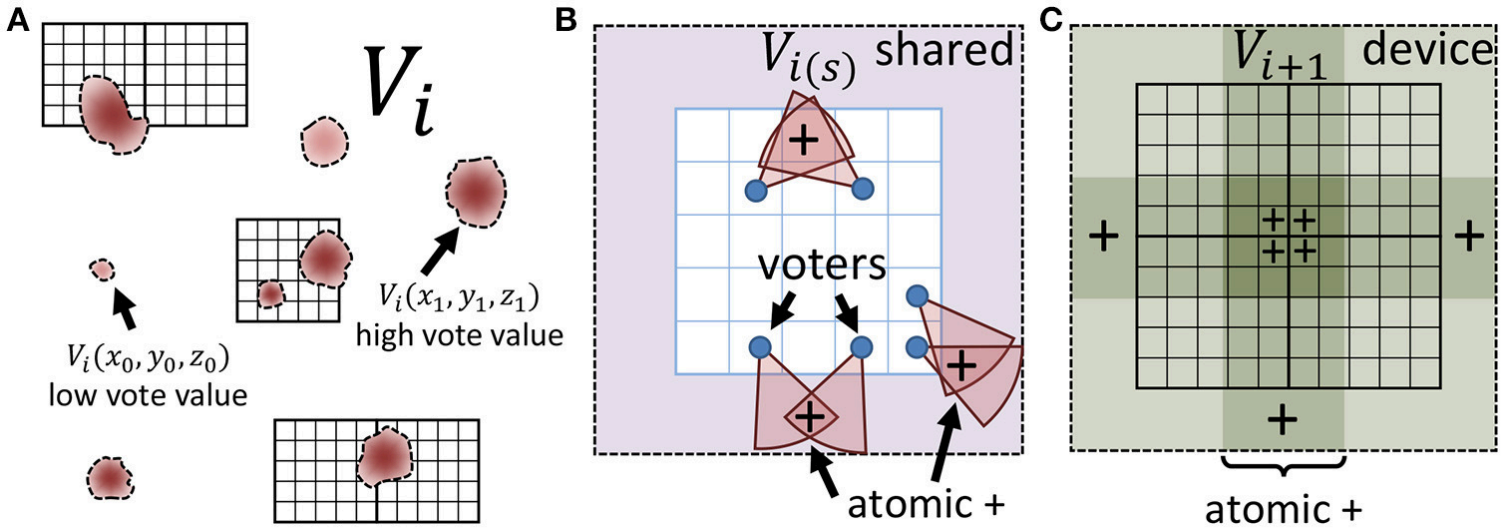
Minimizing memory latency and write conflicts. **(A)** The vote image *V* at iteration *i* is mapped to a CUDA grid composed of square thread blocks. **(B)** Each thread block writes votes to shared memory using atomic adds to ensure that simultaneous votes (+) are correctly cast. **(C)** The shared region for each block is copied to global memory using atomic operations to ensure that votes from adjacent blocks (+) are cast correctly.

When the input is large, or consists of several images (e.g., video), the voting and update steps require a large number of data fetches and calculations, resulting in detection time that cannot keep up with image acquisition. In this section, we show that iterative voting can be highly data parallel, making it amenable to inexpensive (GPU based) acceleration. Instead of using a multi-core CPU, we take advantage of thousands cores in a GPU to speed up the algorithm. Even though CPU cores are more powerful, using thousands GPU cores increases the throughput of the algorithm for volumetric datasets. In addition, each streaming multiprocessor (SM) on a GPU is equipped with a shared memory unit that is close to the processor and therefore decreases memory latency. Atomic memory operations also allow several cores to concurrently access a block of memory by locking access until the operation is completed for each core (Figure 4).

We implemented the proposed algorithm using the CUDA platform using the following kernels:

- **Gradient** - ∇*I* is calculated in parallel using finite differences as a stencil operation. We use an *O*(*h*^2^) calculation (central differences) for central values with *O*(*h*) at the edges. A larger stencil may be more robust, or serve as a replacement for blurring, in high-SNR applications.
- **Voting** - Voting, described in detail below, is implemented as a stencil operation where each thread is assigned a voter and given the task of writing data to *V* within the corresponding vote cone.
- **Direction Update** - The voter direction is updated by tasking each thread to search the corresponding vote cone for the local maximum value and updating the angle in Θ.
- **Maxima** - Local maxima are calculated using a simple stencil operation and collected into a list of point/score pairs using the CPU.

In the first kernel, each thread computes the gradient magnitude and direction, saving the necessary values to *M* and Θ_0_ (Algorithm 1, lines 1–2). The voting kernel (Algorithm 1, lines 8–11) adds the gradient magnitude of each voter to *V* at all pixels within its vote cone. Each voter is assigned a thread that uses Equations (4) and (5) to find the cone. In order to keep the application data parallel, all pixels within an (*r* + 1) × (*r* + 1) window are considered. Note that a large number of pixels outside of the vote cone are considered, particularly as ϕ becomes small. While this must be addressed in the 3D case (section 2.3), there is no significant improvement in 2D unless *r* is extremely large. In that case, down-sampling the image is more practical.

To improve efficiency, Θ was descretized and a look-up table is used for the necessary tan^−1^ calculations (Equation 4). Global memory fetches are reduced by calculating *V* for each block in shared memory. Since each pixel in *V* may be a member of multiple vote cones, atomic additions are required, leading to potential stalls. However, these occur with low probability during early iterations due to the large vote cone coverage. As the probability of a conflict increases with reduced ϕ, the smaller vote cones result in a fewer fetches that offset the reduced occupancy. A third kernel updates voter directions as outlined in Algorithm 1 lines 14–16. Again, each voter is assigned to a thread. Since no writes to *V* will be performed, the vote-cone membership test is optimized by copying the tan^−1^ look-up table to shared memory. Each pixel of *V* within the vote cone is accessed to find the maximum value, which is then used to update Θ(**t**). The final kernel calculates the local maxima of the vote image (Algorithm 1, lines 20, 21) using a stencil of *r*. Local maxima are stored in a list with scores equal to *V* at the corresponding points.

If cell localization is the final desired step, a threshold can be applied based on score using manual or automated (Otsu, 1979) methods. However, cell localization is usually followed up with further segmentation (Merouane et al., 2015), taking the localization score into account as a measure of posterior probability.

### 2.3. Three-dimensional implementation

The principals of three-dimensional iterative voting have been previously explored for cell culture fluorescent images (Han et al., 2011). However, the required number of image fetches limits a single-threaded algorithm to relatively small data sets. Due to the recent availability of large 3D data (Yuan et al., 2015; Xiong et al., 2017), the need has arisen for fast algorithms that can be applied on terabyte-scale data with more complex labeling.

We first reformulate the iterative voting algorithm for efficient implementation in three dimensions. Since the proposed algorithm is orders of magnitude faster than a CPU-based implementation, we limit discussion to the data parallel implementation (Algorithm 2). Modifications from the 2D parallel implementation include:

1. **Cartesian coordinates** - We store the gradient direction using a Cartesian vector field **G**, which allows us to avoid discretization of the 3D spherical vector space to generate look-up tables and increases the efficiency of vote cone membership calculations.
2. **Memory-usage** - We quickly encounter device memory limitations for large-scale data sets. In order to preserve memory, the vector magnitude field *M* is embedded in the Cartesian vector field *G*. As with the 2D algorithm, this allows us to represent the vote magnitude and orientation in the same number of dimensions as the input image. However, additional calculations are required to separate the orientation and magnitude components.
3. **Vote Cone Bounding** - We generate a bounding volume for the vote cone to reduce the number of pixels in *V* considered for membership. This is because the ratio of pixels within the vote cone to those near the voter increases significantly with dimension.

**Algorithm 2:**
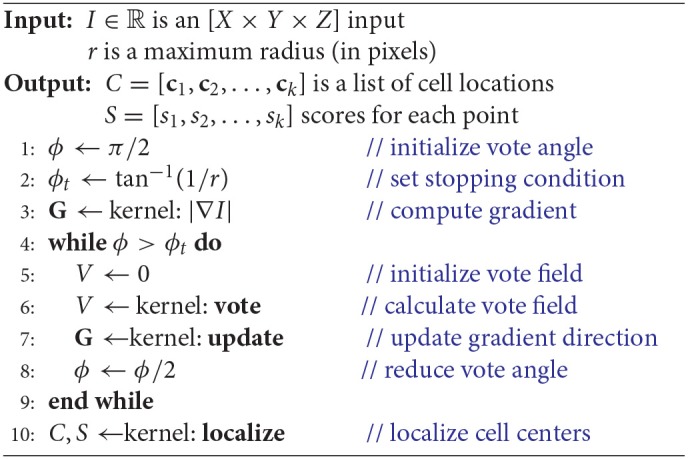
Parallel iterative voting in three dimensions.

The main objective of modifications (1) and (2) is to minimize both computation and memory usage, while both (2) and (3) address the constraints imposed by increasing dimensionality.

The vote angle ϕ and stopping condition (ϕ_*t*_), in this case solid angles, are initialized as explained previously (section 2.1). The first kernel computes the gradient **G** = ∇*I* in Cartesian coordinates (Algorithm 2, line 3), such that **G**(*x, y, z*) ∈ ℝ^3^. This calculation is represented as a separable convolution, and standard methods are used to optimize this calculation using CUDA (Pang et al., 2016). The second kernel calculates the vote field (Algorithm 2, line 6) by assigning a thread to each voter and applying the gradient |*G*| to each pixel within the vote cone. A point **p** is within the vote cone of **t** if it satisfies the following inequalities:

(9) $$\begin{array}{l} \frac{\text{t}-\text{p}}{\left|\text{t}-\text{p}\right|}\cdot \frac{\text{G}\left(\text{t}\right)}{\left|\text{G}\left(\text{t}\right)\right|}>\cos\frac{\phi }{2} \end{array}$$

and

(10) $$\begin{array}{l} |\text{t}-\text{p}|<r \end{array}$$

where **t** is a voter position, and **p** is a pixel position in *V* that receives the vote. Atomic additions are used to sum the vote score in shared memory. The scores for a thread block are copied to global memory after execution.

The third kernel updates **G** to reflect the new voter direction (Algorithm 2, line 7) by using Equations (9) and (10) to find pixels within the vote cone. Valid pixel values in *V* are fetched to find the maximum:

(11) $$\begin{array}{l} {\text{G}}_{i+1}\left(\text{t}\right)=\frac{\left|{\text{G}}_{i}\left(\text{t}\right)\right|}{\left|\text{d}\right|}\text{d} \end{array}$$

where

(12) $$\begin{array}{l} \text{d}=\left[\underset{\text{p}\in \text{cone}}{\text{arg max }}V\left(\text{p}\right)\right]-\text{t} \end{array}$$

Finally, last kernel (Algorithm 2, line 10) localizes cell centers as described in section 2.2.

**Figure 5:**
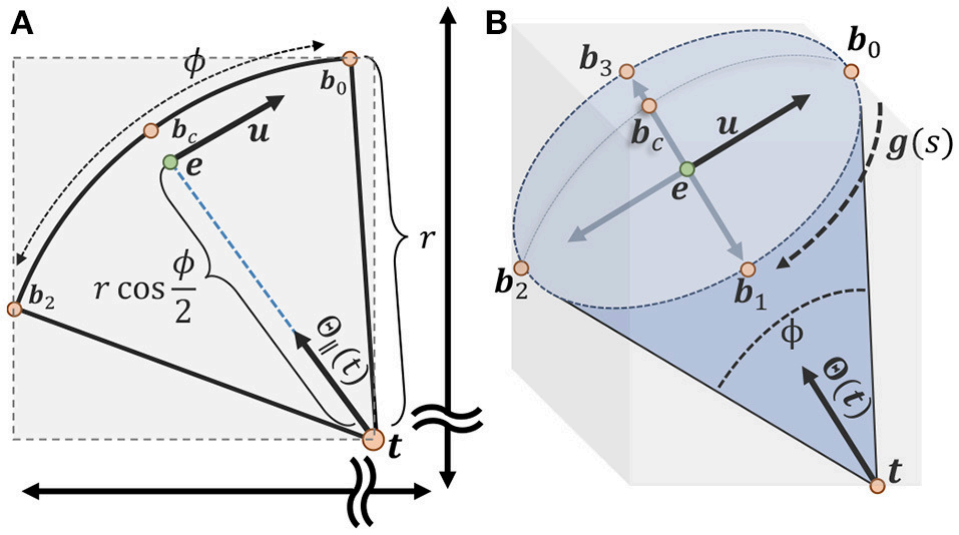
Calculating an axis-aligned bounding box to minimize candidate points for the vote cone. **(A)** The bounding box is initialized with two points t and *b*_*c*_ on either end of the vote cone. The basis vector **u** is used to find the remaining points along the cone ridge. **(B)** All six points used to define the bounding region, with *b*_0_ to *b*_3_ lying on the ridge parameterized by g(s) (more details about calculating the points could be found in supplementary material).

As the vote angle ϕ becomes small, the ratio of accessed candidates within a window around the voter **t** becomes increasingly small, leading to stalls in a parallel implementation. In order to mitigate this problem, we limit the number of candidate points by tightly bounding the vote cone using an axis-aligned bounding box defined by the points:

(13) $$\begin{array}{l} B=\left\{\text{t},{\text{b}}_{c},{\text{b}}_{0},{\text{b}}_{1},{\text{b}}_{2},{\text{b}}_{3}\right\} \end{array}$$

where **t** is the tip of the voter cone and **b**_*c*_ is the furthest candidate point from the voter and corresponds to the center of the spherical cap (Figure 5).

Limiting candidate points to the bounding volume specified by **b**^−^ and **b**^+^ significantly reduces the number of membership tests necessary to both evaluate the vote image and update voter directions. The main benefit is a significant reduction in the number of stalls encountered, resulting in greater GPU occupancy. Without this constraint, almost every test in the local 2*r* + 1 × 2*r* + 1 pixel region will in a memory fetch, stalling execution for several threads. Note that we are using an axis-aligned bounding volume, so the efficiency of the fit is dependent on angle. While a more robust constraint may be possible, the axis-aligned approach is simple to implement and provides a 10X - 100X execution speedup, particularly as ϕ becomes small. Additional details regarding the implementation of the vote cone bounding volume can be found in the Supplementary Material.

## 3. Results

In this section, we demonstrate the effectiveness of the proposed algorithm on two groups of data: (1) nissl stained images collected using KESM, and (2) 3D fluorescent images, including publicly available data sets. In all cases, the only input parameter is the maximum radius *r* in pixels. Since the pixel size of all of the sample images is known, we provide this value in micrometers, which makes our algorithm independent of sampling resolution and anisotropy. If the pixel size is not known, this value must be expressed in pixels.

### 3.1. Cell localization in KESM images

**Figure 6:**
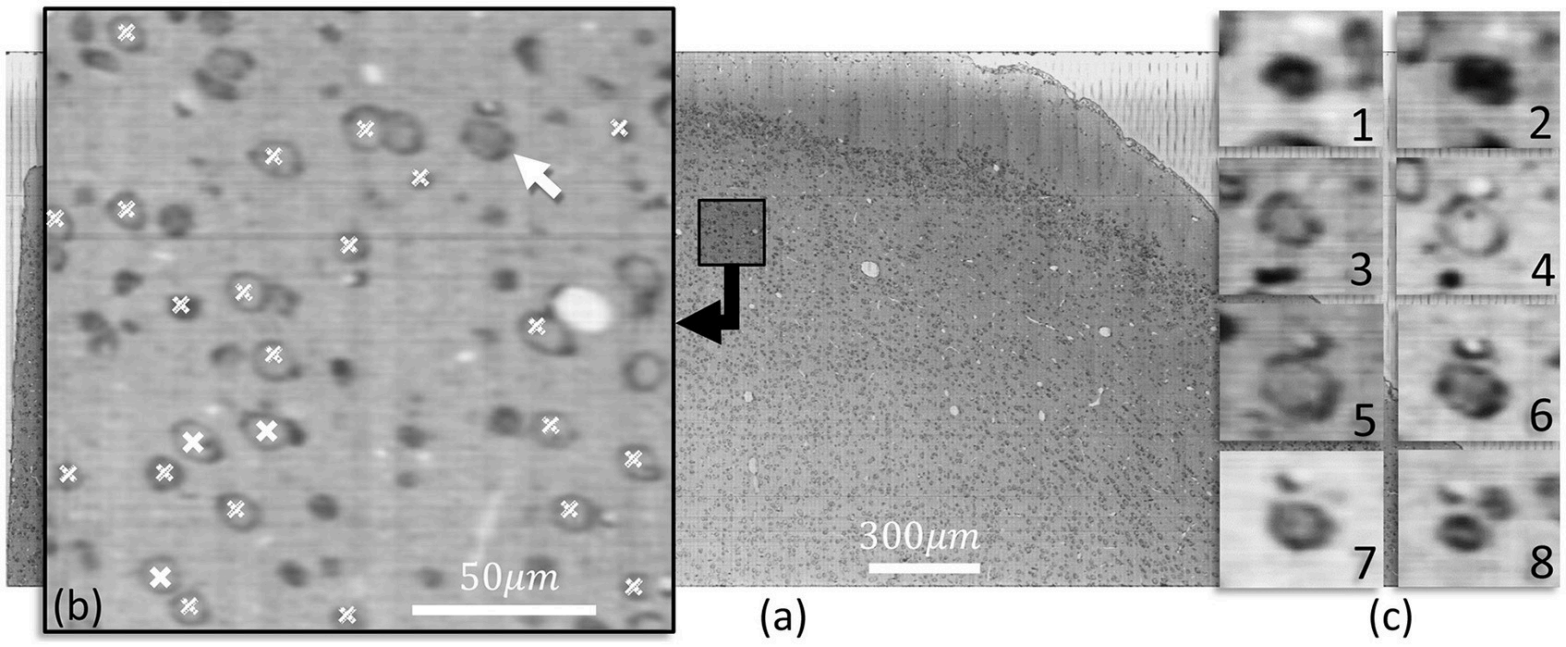
**(a)** A KESM slice of thionin stained mouse cortex is shown along with a close-up **(b)** indicating detected cell positions using iterative voting in the showing slice (white labels) and in two adjacent slices (labels filled with diagonal lines). **(c)** Cross-sections along the z-axis of a neurons indicated with an arrow in **(b)** are shown, highlighting the structure. The nucleolus of the cell are visible in c-4, the same slice that is detected by the proposed algorithm.

Figure 6 shows a slice of nissl-stained mouse cortex imaged using KESM. Cells detected using the proposed method are indicated in a closeup section. The three dimensional structure for one neuron is shown along the *z* axis.

**Figure 7:**
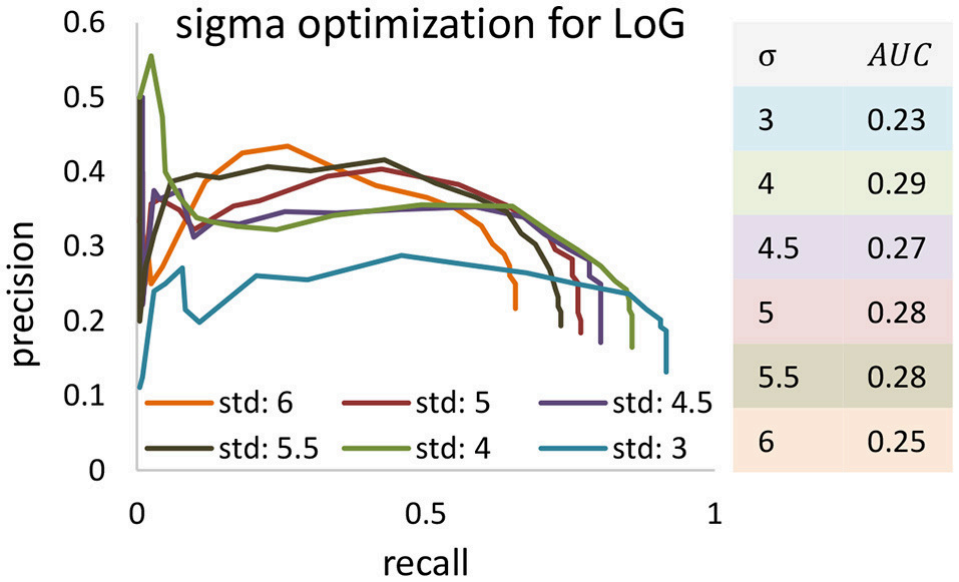
The precision recall curve for applying state of the art and iterative voting algorithms on the KESM dataset. Some pre-processing steps are applied on the KESM datasets to get best results. Intensity provided by some of algorithms are used to generate precision recall curves.

Precision-recall curves are used to compare results produced by the proposed algorithm (ivote3) to several cell localization methods and software packages, including FARSIGHT (Al-Kofahi et al., 2010), MINS (Lou et al., 2014), 3D object counter plug-in imageJ (Bolte and Cordelières, 2006), Laplacian of Gaussian (LoG) filter (Marr and Hildreth, 1980), 3D-MLS (Chinta and Wasser, 2012), and LoS (Mathew et al., 2015) (Figure 7).

**Figure 8:**
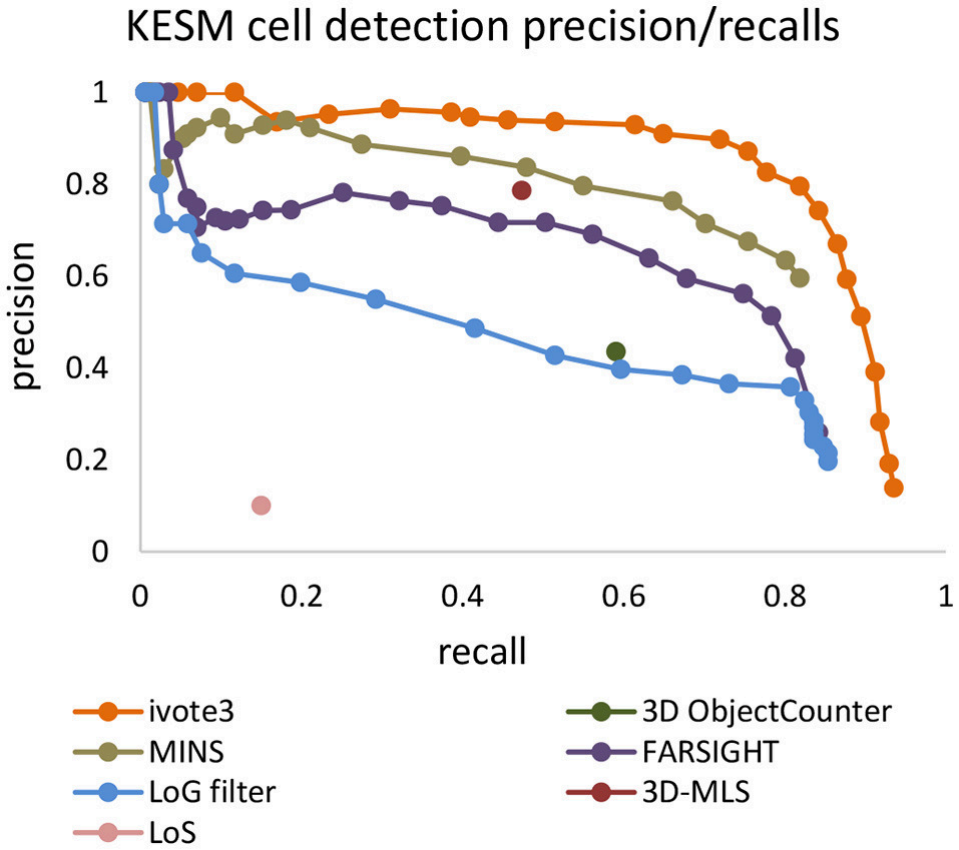
Comparing validation results for 3D iterative voting and LoG implementations for blob detection of KESM dataset. Average values of precision and recall features for four datasets are computed, which are enveloped by standard deviations.

LoG blob detection is frequently used for localization, and several of the tested algorithms utilize LoG filtering as a pre-processing step (Al-Kofahi et al., 2010). However, our experiments indicate that these types of dense data result in LoG performance that is highly sensitive to input parameters (Figure 8). The proposed ivote2 and ivote3 algorithms mitigate much of this sensitivity with very little reduction in performance, even when compared to a parallel LoG implementation.

**Figure 9:**
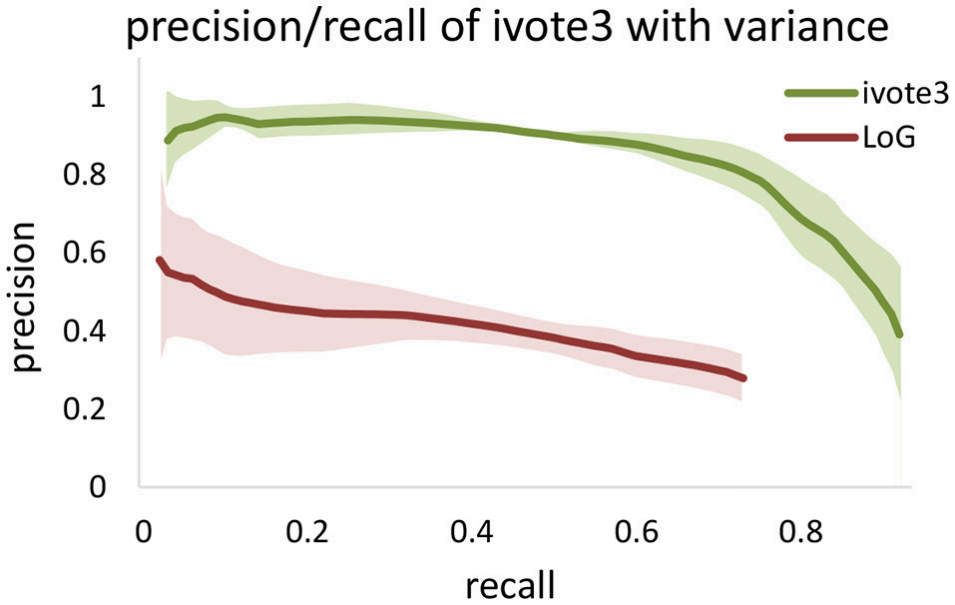
The precision-recall curve for applying the Laplacian of Gaussian method for blob detection in KESM dataset. Different values are set to Gaussian standard deviation to find the best validation result. Area under curve are computed and based of that standard deviation of four was selected.

We also tested performance across multiple data sets, given that optimal segmentation parameters tend to vary across large images. We manually segmented 4,512^3^ cubes from the KESM dataset and compared the variance in performance for both ivote3 and LoG filter (Figure 8). The variance for LoG uses optimal parameters selected from Figure 9.

### 3.2. Cell localization in 3D confocal microscopy

We demonstrate the effectiveness of our algorithm on several fluorescent data sets. Two data sets were acquired from publicly available sources and demonstrate the viability of parallel iterative voting on traditional images. We also acquired a larger-scale 3D confocal data set to demonstrate the benefit of our method on large-scale images using traditional fluorescence microscopy:

- **Confocal microscopy/ GFP transfection stained:** (Maška et al., 2014; Ulman et al., 2017), available at celltrackingchallenge.net (Fluo-N3DH-CE).
- **Confocal microscopy/ Hoechst stained:** (Maška et al., 2014; Ulman et al., 2017), available at celltrackingchallenge.net (Fluo-N3DH-SIM+).
- **Confocal microscopy/ DAPI stained:** The hilus region of the dentate gyrus in the mouse hippocampus was imaged using a 40*X* oil objective on a Leica TCS SP8 confocal microscope. The DAPI signal was excited by a 405nm laser. Acquisition speed was set to 600 Hz, with a 0.75 zoom factor. Raw images for all data analysis were exported as TIFFs. Transgenic mice that model Dravet syndrome with spontaneous seizure onset at postnatal day 15 were housed in a 12 hour light/dark cycle. These mice have a knock-in mutant Scn1A gene containing a nonsense substitution (CgG to TgA) in exon 21 (Ogiwara et al., 2007). All animal experiments were approved by the Institutional Animal Care and Use Committee of the University of Houston. This method was used to create two datasets: one from adult mice (mouse-HPC), and the other one from postnatal day 11 mouse pups (mouse-HPC.11).

**Figure 10:**
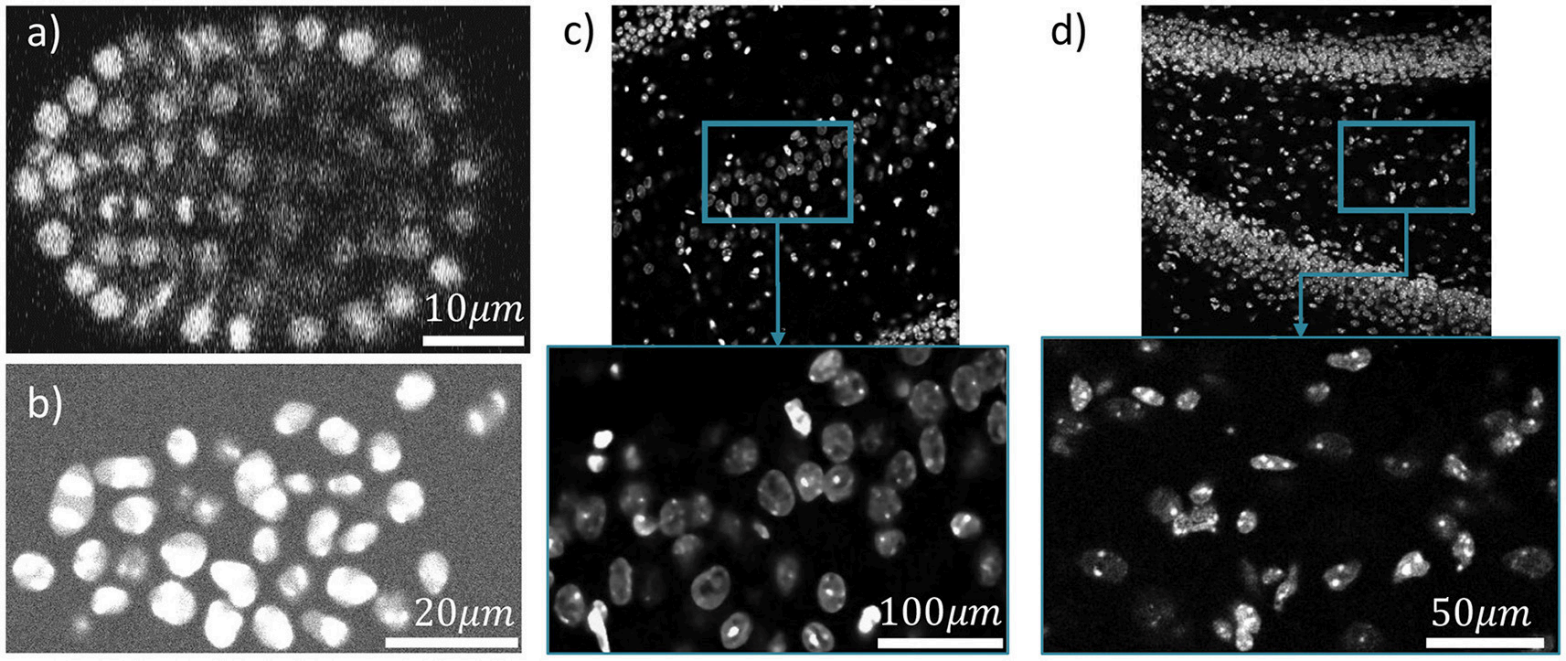
Four different datasets are used for cell localization using ivote3. One slice of them along z axis is shown **(a)** *C. elegan* developing embryo (Flou-N3DH-CE). **(b)** Simulated nuclei of HL60 cells (Flou-N3DH-SIM+). **(c)** The hilus region of the dentate gyrus in the adult mouse hippocampus (mouse-HPC). **(d)** The hilus region of the dentate gyrus in the day 11 mouse hippocampus (mouse-HPC.11).

**Figure 11:**
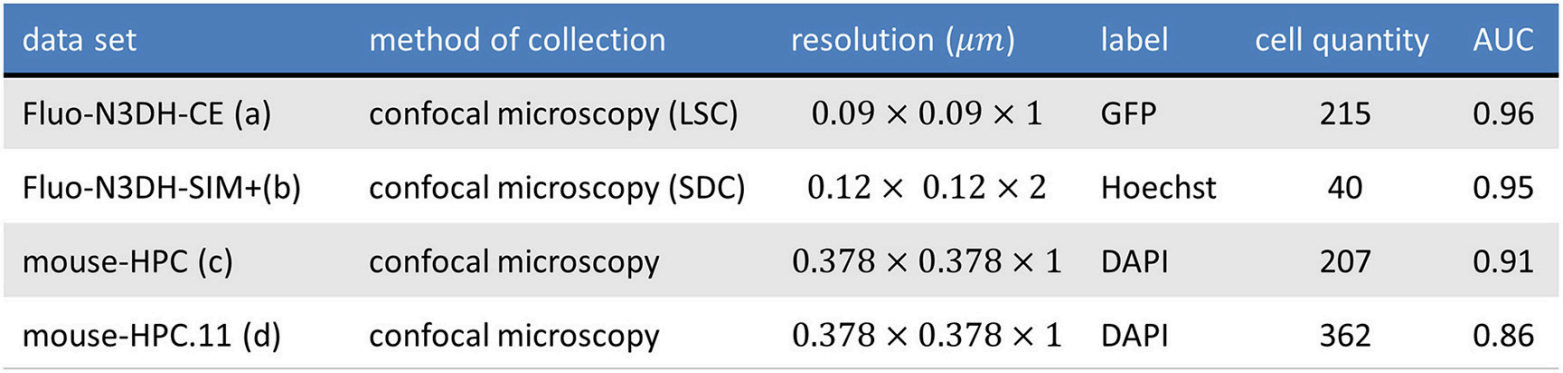
The table shows the information of tested datasets including name, method of collection, resolution, label, number of annotated cells, and area under precision recall curve of applying ivote3 method.

Figure 10 shows one slice of each dataset along the *z* axis. Information about these datasets is shown in Figure 11. Area under curve (AUC) is calculated from the precision recall curve of the proposed algorithm.

**Figure 12:**
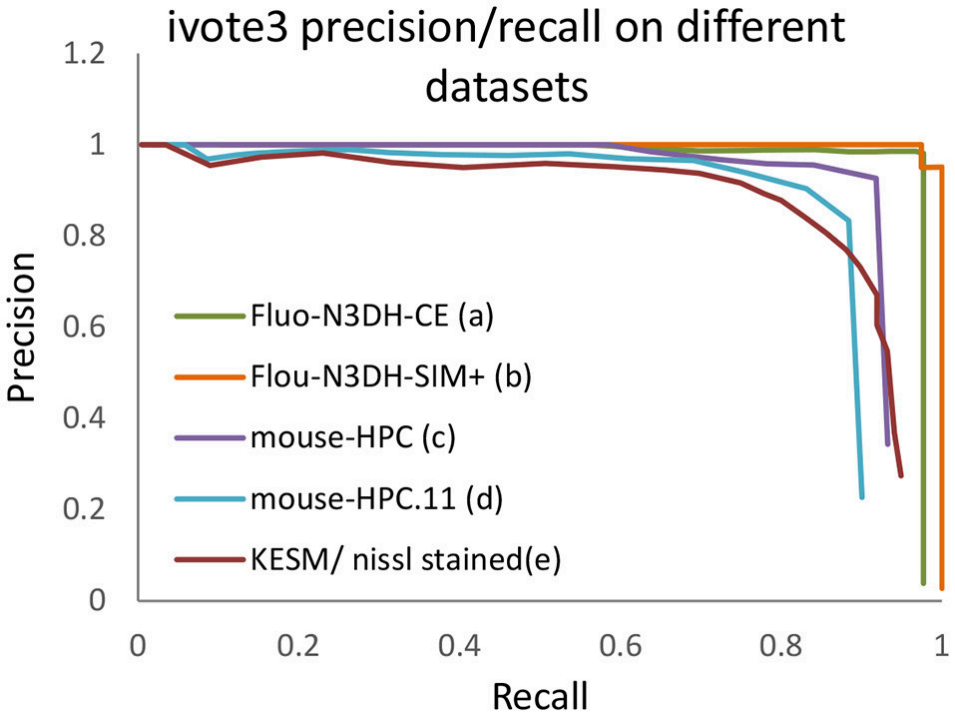
The precision recall curve for applying the iterative voting algorithm on different datasets. A detected cell is considered as a true positive (TP) if its distance of an annotated nuclei is less than or equal to fifty percent of maximum radius.

As a pre-processing step, we use the Gaussian kernel to blur these datasets and then localize the cells using the proposed algorithm. The only parameter that has to be set is maximum radius of cells in pixels. A ground truth is manually annotated for each dataset, which includes the location of cells, to validate localization results. Figure 12 indicates the validation results using the proposed algorithm for cell detection on different datasets.

### 3.3. Profiling

In this section, we extensively profile the proposed algorithm, and discuss current performance limitations. These optimizations open several doors for 2D image processing at a large scale, and are critical for 3D localization.

Our algorithm was implemented on a nVvidia GeForce (GTX 970) with 1664 CUDA cores, 4*GB* of global memory, 1.75*MB* of *L*2 cache size, and 48*kB* of on-chip shared memory. The compute capability is 5.2, the global memory bandwidth is 224.32*GB*/*s*, and the single precision FLOP/s is 4.423*TeraFLOP*/*s*.

**Figure 13:**
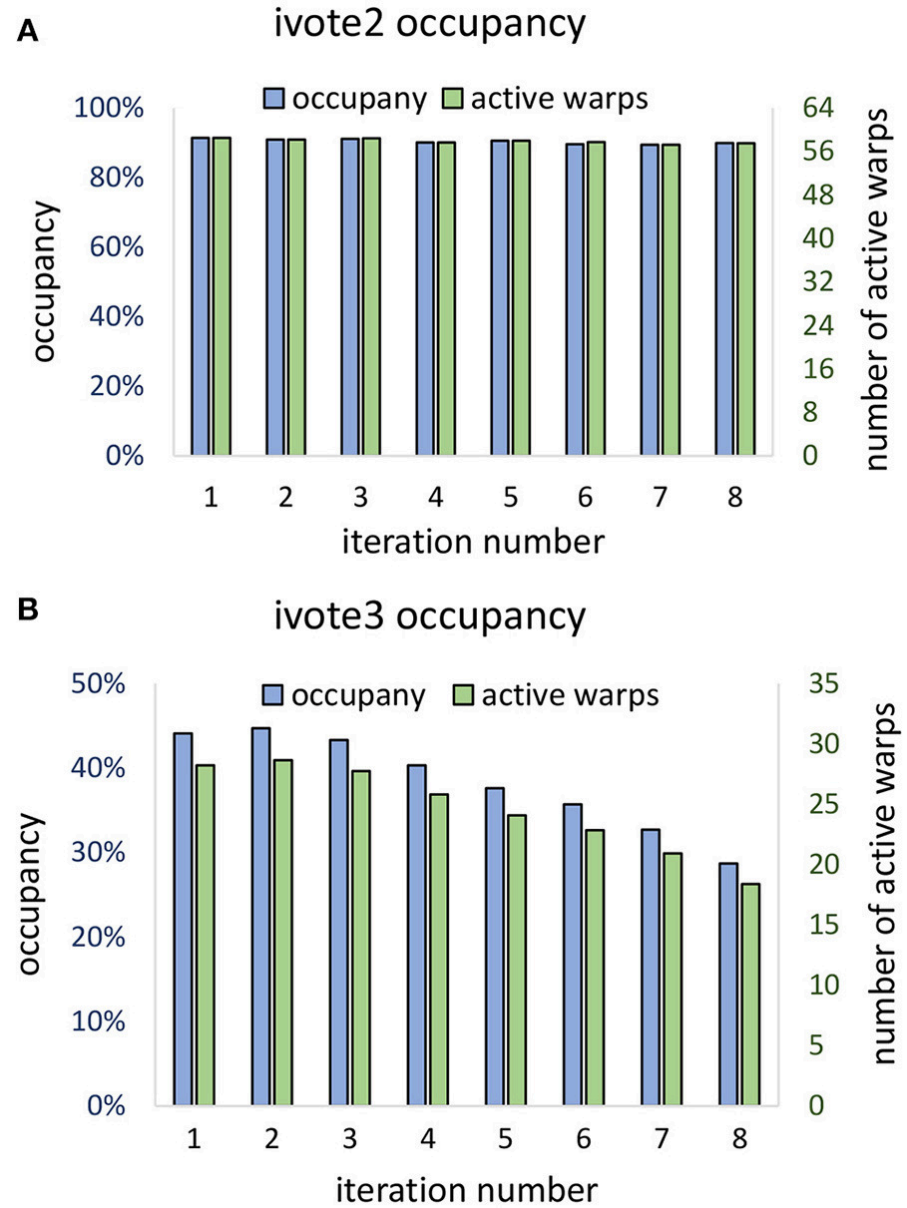
Vote kernel occupancy is shown for **(A)** two dimensional (ivote2) and **(B)** three dimensional (ivote3) implementation. In ivote2, occupancy and active warps are consistent during iterations, suggesting that there are very few stalls due to atomic writes. In ivote3 the occupancy falls with consecutive iterations, leading to reduced occupancy (≈ 10%). This is due to an increased number of write conflicts with higher dimension and increased latency since writes are applied directly to global memory. Note that the theoretical occupancy decreases from 100% (ivote2) to 50% (ivote3) due to increased register use.

Theoretical occupancy of 50% is limited by the number of registers (46) used per thread. Our algorithm was able to achieve ≈ 44% occupancy during the first iteration, which dropped off during consecutive iterations (Figure 13B). This falloff is likely due to the increase in stalls due to shared memory write conflicts caused by the required atomic addition. Consecutive iterations result in a reduced ϕ and a vote cone more likely to overlap with adjacent voters. In two dimensions, theoretical occupancy is 100%, and ≈ 90% was achieved by the proposed algorithm. Since vote cones have less members, there is lower chance of writing conflicts, and so active warps and occupancy are not effected by the iteration number (Figure 13A).

**Figure 14:**
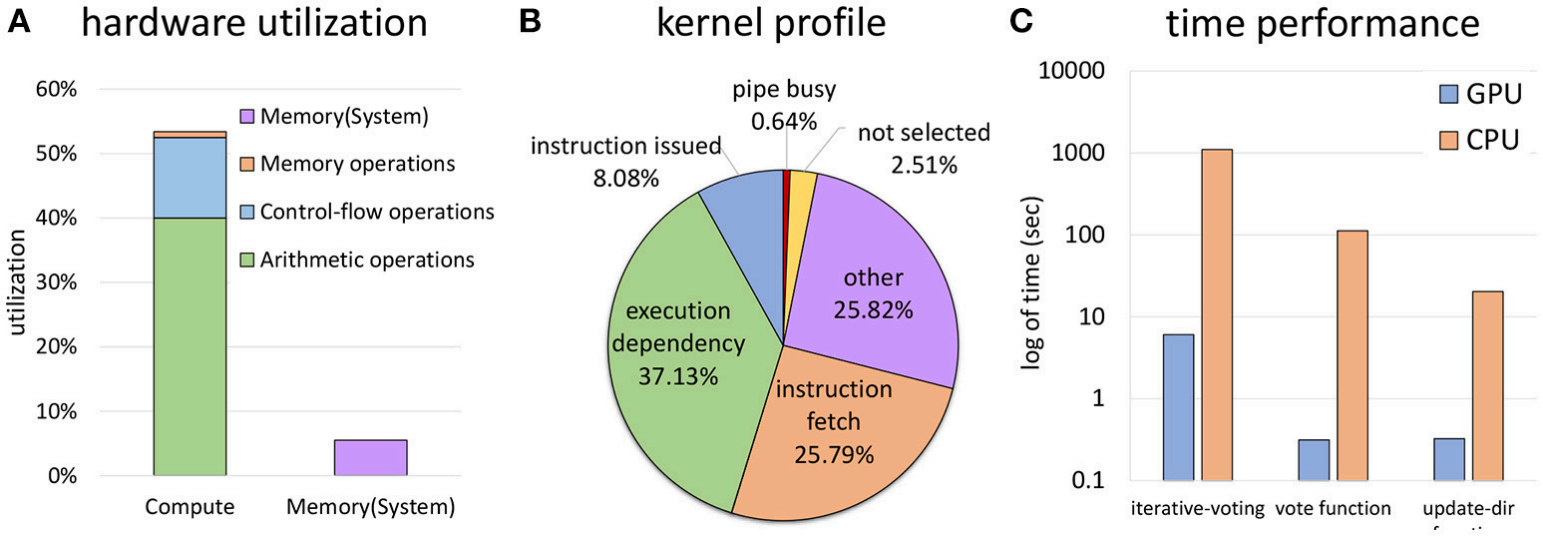
Computation due to a huge number of vote cones calculations at each thread is the bottleneck for ivote3 **(A)**. Using bounding volume improved time performance and decreases branch divergence, but the main reason of stalls still is thread divergence **(B)**. Time performance is significantly improved through data parallel in comparison with a highly optimized CPU implementation **(C)**.

Given the theoretical occupancy, our algorithm is primarily compute limited, owing to a large number of vote cone calculations (Figures 14A,B). A large number of stalls are due to execution dependencies within warps, largely due to thread divergence. The use of a bounding volume for the cone (section 2.3) was largely done to mitigate these stalls and further tightening of this volume could significantly increase performance.

Overall performance shows a significant speedup of 2–3 orders of magnitude over a highly optimized CPU implementation (Figure 14C) using a consumer nVidia GeForce GTX 970, making this code practical for 3D images. The ivote3 algorithm is also fully parallizable, allowing the data set to be split across multiple GPU co-processors as necessary.

## 4. Discussion and future work

Other approaches were tested to improve performance. In particular, the gradient vector flow (GVF) method (Xu and Prince, 1997) was implemented to replace the blurring step. While we expected better accuracy, the final results did not show any improvement beyond blurring for the KESM dataset. Our next approach will focus on the use of a convolutional neural network (CNN) (Lee et al., 2015; Apthorpe et al., 2016; Zlateski et al., 2017) to replace the blurring step. Since the cell types in this mouse cortical data set are highly variable and not always rotationally symmetric, we expect a trained pre-processing method to produce structures that are more readily localized. In addition, the resulting features may be utilized to automatically identify cell types. Methods for classifying cells based on local features is also promising, since recent advances in KESM imaging may allow collection of up to 3 channels of multispectral data (Zheng et al., 2013). We also expect that CNNs will provide a robust set of features that can be used for classification.

We see two areas where performance improvements are most likely: increasing the theoretical occupancy and reducing thread divergence. Theoretical occupancy is limited by the number of available registers, and an increased register file size could potentially increase the number of threads that can be executed simultaneously. Alternative hardware, such as newer Pascal GPU architectures which support single-precision (16-bit) floating point (Ho and Wong, 2017), may provide a significant advantage since iVote does not require 32-bit processing. Tests on alternative architectures, such as the Intel Xeon Phi, may yield better results in terms of thread divergence since they are more robust to these issues than the CUDA SIMD warp-based architecture.

## 5. Conclusion

In this paper, we propose several advances in iterative voting to make it more applicable to large-scale 2D and 3D images. We re-formulate several components of the algorithm in order to significantly reduce the required input parameters, thereby reducing the need for human intervention during computation. We also propose a data parallel formulation that provides accurate localization results in real-time image processing. Finally, we extend the iterative voting algorithm into three-dimensional images, which is only practical using our data parallel framework. Our software exhibits localization performance superior to all of the 3D algorithms that we've tested, and is the only algorithm we've found that performs acceptably on dense and feature-rich images such as those acquired using KESM. Validation results demonstrate that iterative voting works well for detecting cell nuclei in volumetric datasets with varying size and shape, and it is a robust algorithm where cells are densely packed, which is often seen in KESM brain data.

Profiling results indicate the efficiency of the algorithm which is practical for big images, videos, and volumetric dataset. Minimal user input and no user feedback, making this algorithm amenable to fully automated processing of terabyte-scale data sets. We believe that this framework is particularly suitable as a first-pass localization step for several segmentation algorithms. We are currently exploring applications in improving performance in 2D large-scale images acquired using TIMING (Merouane et al., 2015) as well as cell identification and segmentation in KESM images.

## Author contributions

LS and DM: designed and developed the proposed algorithm; LA: designed and performed KESM labeling; DM: collected KESM images; LM and JZ: developed the labeling methods and collected confocal data sets. All authors have contributed to revising the manuscript.

### Conflict of interest statement

The authors declare that the research was conducted in the absence of any commercial or financial relationships that could be construed as a potential conflict of interest.
