# Supplementary material for: Robust Cell Detection for Large-Scale 3D Microscopy Using GPU-Accelerated Iterative Voting
Source: Front Neuroanat. 2018 Apr 26;12:28. doi: 10.3389/fnana.2018.00028 (PMC5932171; doi:10.3389/fnana.2018.00028)
Supplement: Supplementary file 1 [file Presentation_1.PDF]

Supplementary Material: Robust Cell Detection for Large-Scale 3D Microscopy Using GPU-Accelerated Iterative Voting

TWO DIMENSIONAL LOCALIZATION

Two-dimensional localization results are demonstrated on histological data sets. The first example shows localization of nuclei labeled positive for estrogen receptor (ER) in a breast tumor biopsy (Figure S1(a)). The tissue sample was obtained from a commercial tissue micro-array (Biomax, Inc.) and stained for ER. The second sample is human liver labeled using hematoxylin and eosin (H&E) (Figure S1(b)). The proposed methods make obtaining accurate cell counts practical for large volumes consisting of hundreds to thousands of 2D sections similar to those currently used by pathologists.

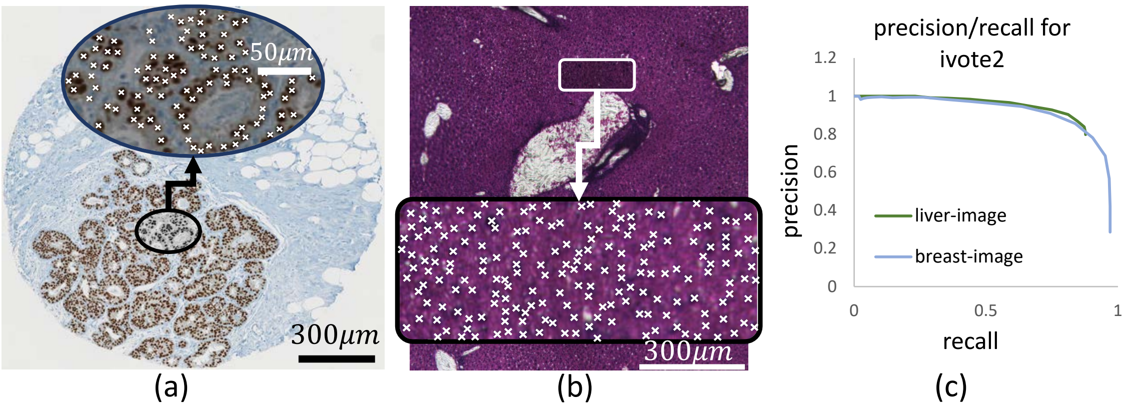

Figure S1: 2D iterative voting is used to localize (a) ER+ epithelial cells in a breast biopsy sample and (b) Human liver nuclei stained with hematoxylin and eosin (H&E). (c) The precision-recall curve for 2D localization is also shown.

VOTE CONE BOUNDING VOLUME

The vote cone bounding volume is defined to include all points in the set $B = \{\mathbf{t}, \mathbf{b}_c, \mathbf{b}_0, \mathbf{b}_1, \mathbf{b}_2, \mathbf{b}_3\}$, where $\mathbf{b}_c = \mathbf{t} + r\boldsymbol{\Theta}(\mathbf{t})$ and $\boldsymbol{\Theta}(\mathbf{t})$ is the end cap normal. The next four points around the end cap are calculated using the parametric expression:

$$g(s) = r\cos(s)\mathbf{u} + r\sin(s)\boldsymbol{\Theta}(\mathbf{s}) \times \mathbf{u} + \mathbf{e} \tag{S1}$$

where $\mathbf{e}$ is the end cap center $\mathbf{e} = \mathbf{t} + r\boldsymbol{\Theta}(\mathbf{t})\cos\left(\frac{\phi}{2}\right)$, and $\mathbf{u}$ is a basis vector parameterizing the edge of the end cap. We calculate $\mathbf{u}$ by first defining $\boldsymbol{\Theta}_{\|}(\mathbf{t}) = [\Theta(\mathbf{t})_x \quad \Theta(\mathbf{t})_y]^T$ as the projection of the voter direction $\boldsymbol{\Theta}(\mathbf{t})$. We then rotate $\boldsymbol{\Theta}_{\|}(\mathbf{t})$ by 90-degrees about the z-axis and normalize to calculate $\mathbf{u}$:

$$\mathbf{u} = \frac{1}{|\boldsymbol{\Theta}_{\|}(\mathbf{t})|}\begin{bmatrix} 0 & -1 \\ 1 & 0 \end{bmatrix}\boldsymbol{\Theta}_{\|}(\mathbf{t}) = \frac{1}{|\boldsymbol{\Theta}_{\|}(\mathbf{t})|}\begin{bmatrix} -\Theta(\mathbf{t})_y \\ \Theta(\mathbf{t})_x \end{bmatrix} \tag{S2}$$

If the length of $\boldsymbol{\Theta}_{\|}(\mathbf{t})$ equals to zero, $\boldsymbol{\Theta}(\mathbf{t})$ is aligned along the z-axis and $\mathbf{u} = [1 \quad 0]^T$. The remaining points in B can then be calculated by sampling the parametric function: $\mathbf{b}_0 = g(0)$, $\mathbf{b}_1 = g\left(\frac{\pi}{2}\right)$, $\mathbf{b}_2 = g(\pi)$, $\mathbf{b}_3 = g\left(\frac{3\pi}{4}\right)$.
